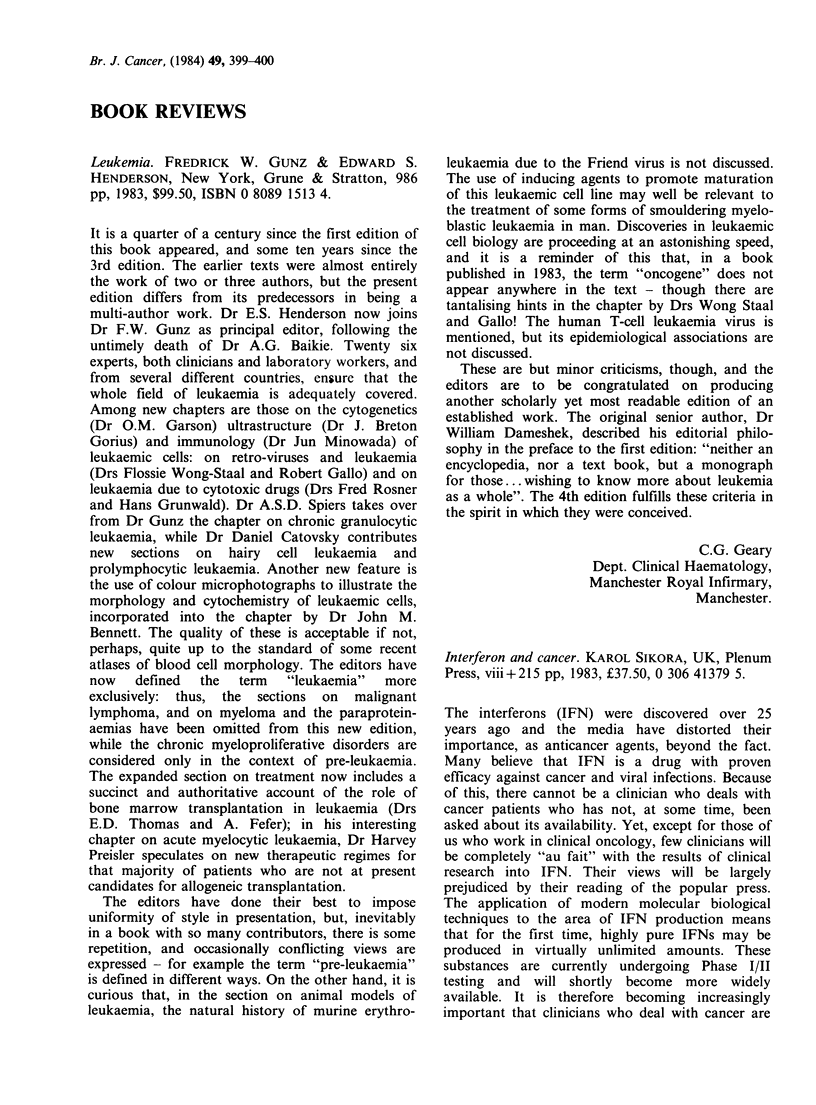# Leukemia

**Published:** 1984-03

**Authors:** C.G. Geary


*Br. J. Cancer*, (1984) **49**, 399–400

### BOOK REVIEWS

*Leukemia*. FREDRICK W. GUNZ & EDWARD S. HENDERSON, New York, Grune & Stratton, 986 pp, 1983, $99.50, ISBN 0 8089 1513 4.

It is a quarter of a century since the first edition of this book appeared, and some ten years since the 3rd edition. The earlier texts were almost entirely the work of two or three authors, but the present edition differs from its predecessors in being a multi-author work. Dr E.S. Henderson now joins Dr F.W. Gunz as principal editor, following the untimely death of Dr A.G. Baikie. Twenty six experts, both clinicians and laboratory workers, and from several different countries, ensure that the whole field of leukaemia is adequately covered. Among new chapters are those on the cytogenetics (Dr O.M. Garson) ultrastructure (Dr J. Breton Gorius) and immunology (Dr Jun Minowada) of leukaemic cells: on retro-viruses and leukaemia (Drs Flossie Wong-Staal and Robert Gallo) and on leukaemia due to cytotoxic drugs (Drs Fred Rosner and Hans Grunwald). Dr A.S.D. Spiers takes over from Dr Gunz the chapter on chronic granulocytic leukaemia, while Dr Daniel Catovsky contributes new sections on hairy cell leukaemia and prolymphocytic leukaemia. Another new feature is the use of colour microphotographs to illustrate the morphology and cytochemistry of leukaemic cells, incorporated into the chapter by Dr John M. Bennett. The quality of these is acceptable if not, perhaps, quite up to the standard of some recent atlases of blood cell morphology. The editors have now defined the term "leukaemia" more exclusively: thus, the sections on malignant lymphoma, and on myeloma and the paraproteinaemias have been omitted from this new edition, while the chronic myeloproliferative disorders are considered only in the context of pre-leukaemia. The expanded section on treatment now includes a succinct and authoritative account of the role of bone marrow transplantation in leukaemia (Drs E.D. Thomas and A. Fefer); in his interesting chapter on acute myelocytic leukaemia, Dr Harvey Preisler speculates on new therapeutic regimes for that majority of patients who are not at present candidates for allogeneic transplantation.

The editors have done their best to impose uniformity of style in presentation, but, inevitably in a book with so many contributors, there is some repetition, and occasionally conflicting views are expressed – for example the term "pre-leukaemia" is defined in different ways. On the other hand, it is curious that, in the section on animal models of leukaemia, the natural history of murine erythroleukaemia due to the Friend virus is not discussed. The use of inducing agents to promote maturation of this leukaemic cell line may well be relevant to the treatment of some forms of smouldering myeloblastic leukaemia in man. Discoveries in leukaemic cell biology are proceeding at an astonishing speed, and it is a reminder of this that, in a book published in 1983, the term "oncogene" does not appear anywhere in the text – though there are tantalising hints in the chapter by Drs Wong Staal and Gallo! The human T-cell leukaemia virus is mentioned, but its epidemiological associations are not discussed.

These are but minor criticisms, though, and the editors are to be congratulated on producing another scholarly yet most readable edition of an established work. The original senior author, Dr William Dameshek, described his editorial philosophy in the preface to the first edition: "neither an encyclopedia, nor a text book, but a monograph for those... wishing to know more about leukemia as a whole". The 4th edition fulfills these criteria in the spirit in which they were conceived.

C.G. Geary
Dept. Clinical Haematology,
Manchester Royal Infirmary,
Manchester.